# Genome mining in *Trichoderma viride* J1-030: discovery and identification of novel sesquiterpene synthase and its products

**DOI:** 10.3762/bjoc.15.202

**Published:** 2019-08-28

**Authors:** Xiang Sun, You-Sheng Cai, Yujie Yuan, Guangkai Bian, Ziling Ye, Zixin Deng, Tiangang Liu

**Affiliations:** 1 Key Laboratory of Combinatorial Biosynthesis and Drug Discovery, Ministry of Education and School of Pharmaceutical Sciences, Wuhan University, Wuhan, 430071, P. R. China

**Keywords:** genome mining, metabolic engineering, natural products, sesquiterpene synthase, terpenes, *Trichoderma viride* J1-030

## Abstract

Sesquiterpene synthases in *Trichoderma viride* have been seldom studied, despite the efficiency of filamentous fungi for terpenoid production. Using the farnesyl diphosphate-overexpressing *Saccharomyces cerevisiae* platform to produce diverse terpenoids, we herein identified an unknown sesquiterpene synthase from *T. viride* by genome mining and determined the structure of its corresponding products. One new 5/6 bicyclic sesquiterpene and its esterified derivative were characterised by GC–MS and 1D and 2D NMR spectroscopy. To the best of our knowledge, this is the first well-identified sesquiterpene synthase from *T. viride* to date.

## Introduction

Terpenoids represent the most diverse group of natural products, with a wide distribution in microorganisms, plants, insects and various marine invertebrates [1–2]. More than 80,000 terpenoids have been identified and characterised [3–5]. These diverse and complex natural products are mostly derived from carbocation cyclisation with linear C5 isoprene precursors, which are catalysed by terpene synthases (TPSs) [6]. TPSs can be classified into three types based on their amino acid sequence. Type I TPSs are metal-dependent enzymes that initiate cyclisation by the elimination of diphosphate groups from precursors and carbocation formation, and type II TPSs initiate the catalytic process by the protonation of an olefinic double bond [7]. The recently reported type III TPSs, UbiA-related TPSs, also catalyse cascade reactions by diphosphate elimination [8]. In addition, each type of TPS is characterised by a unique aspartate-rich motif; most type I TPSs have a DDXXD/E motif and an NSE/DTE motif, whereas type II TPSs have the DXDD motif [9–10].

The C_15_ sesquiterpenoids constitute a large class of terpenoids with a wide range of industrial and commercial applications, including uses in flavours and perfumes, as bioactive molecules in the pharmaceutical industry, and in health care products [11]. Sesquiterpenoids are biosynthesised from the universal linear precursor farnesyl pyrophosphate (FPP) and assembled by FPP synthases, using dimethylallyl diphosphate (DMAPP) and isopentenyl diphosphate (IPP) as substrates. The subsequent elimination of diphosphate from FPP is catalysed by sesquiterpene synthases, with further cyclisation steps to form structurally diverse (poly)cyclic core skeletons [3,12]. A set of post-modification enzymes can transform core sesquiterpene skeletons into different kinds of sesquiterpenoids with potential anticancer, cytotoxic and antibiotic functions [13]. More than 121 skeleton structures derived from the sesquiterpene precursor FPP via sesquiterpene synthase have been described. Nearly 75% of these structures have at least one six-membered ring; 69% of these contain five-membered rings, occupying a large portion. Three- and seven-membered ring structures account for just 21% and 24% of these structures, respectively. Four (10%) and eight (7%) membered ring structures (e.g. asteriscanolide) are seldom found [14]. With the lower costs of gene sequencing, recent developments in genome mining by sequencing and annotation have led to the discovery of a large number of functionally unknown terpene synthases [15–17], generating diverse complex structures and several bioactive products (e.g., 6α,9α,15-trihydroxycadinan-4-en-3-one, (+)-3,11,12-trihydroxycalamenene, and (−)-3,10,11,12-tetrahydroxycalamenene) [18].

Filamentous fungi are powerful producers of terpenoid products [19]. Many terpenoids produced by these fungi have recently been characterised; these terpenoids exhibit diverse complex structures and uncommon catalytic mechanisms [20]. However, a limited number of sesquiterpenes have been characterised from a few fungal taxa (e.g., trefolane A and sterhirsutins) [21]. *Trichoderma viride* is a filamentous fungus that has received considerable attention as an effective biocontrol agent against two fungal pathogens, *Fusarium oxysporum* f. sp. *adzuki* and *Pythium arrhenomanes*, infecting soybean. This fungus is a competent mycoparasite and strong producer of secondary metabolites [22–23]. However, *T. viride* terpenoids have rarely been studied and the low concentrations of products under natural conditions have limited the pace of research in this field. Metabolic engineering makes the overproduction of different terpenoids from *T. viride* possible [21,24]. To increase the discovery efficiency of terpenoid products, heterologous expression of various sources of terpene synthases in *Escherichia coli* and *Saccharomyces cerevisiae* is a feasible approach [25–26].

**Figure 1:**
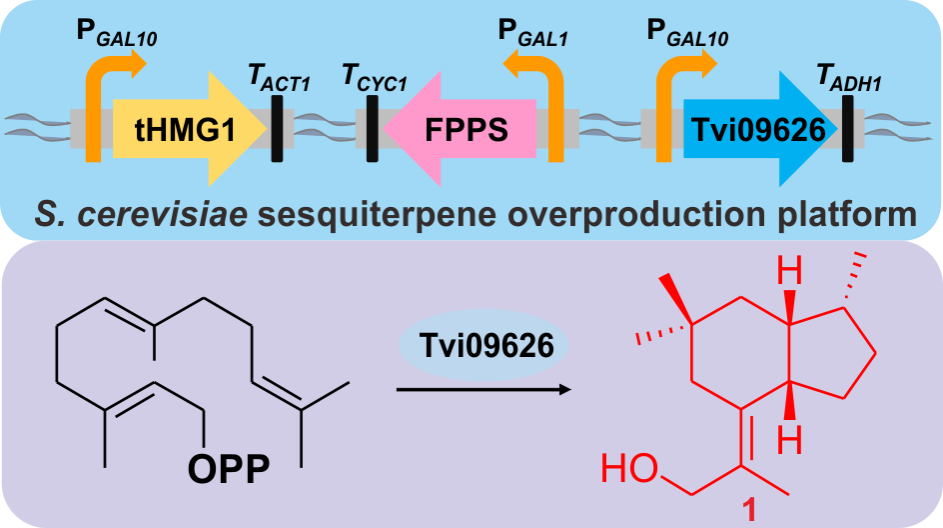
Schematic diagram of the *S. cerevisiae* sesquiterpene overproduction platform and the products of Tvi09626.

In this study, a combination of genome mining and metabolic engineering was used for sesquiterpenoid discovery, utilizing farnesyl diphosphate-overexpressing *S. cerevisiae* as a platform (Figure 1). By the heterologous expression of predicted terpene synthases from the genome of *T. viride*, an unknown sesquiterpene synthase was identified and characterised. Furthermore, a new compound produced by this enzyme and its esterified product were detected and characterised by GC–MS and 1D and 2D NMR, revealing a 5/6 bicyclic sesquiterpene and its C-11 esterified structure. Based on a literature search, to our knowledge, this is the first report of the characterisation of a sesquiterpene synthase in *T. viride*. In addition, this study demonstrates the effectiveness of the combination of genome mining and heterologous expression of predicted terpene synthases for detecting unknown terpenoids from rarely studied fungi.

## Results and Discussion

### Prediction and analysis of terpene synthase genes in *T. viride* J1-030

**Figure 2:**
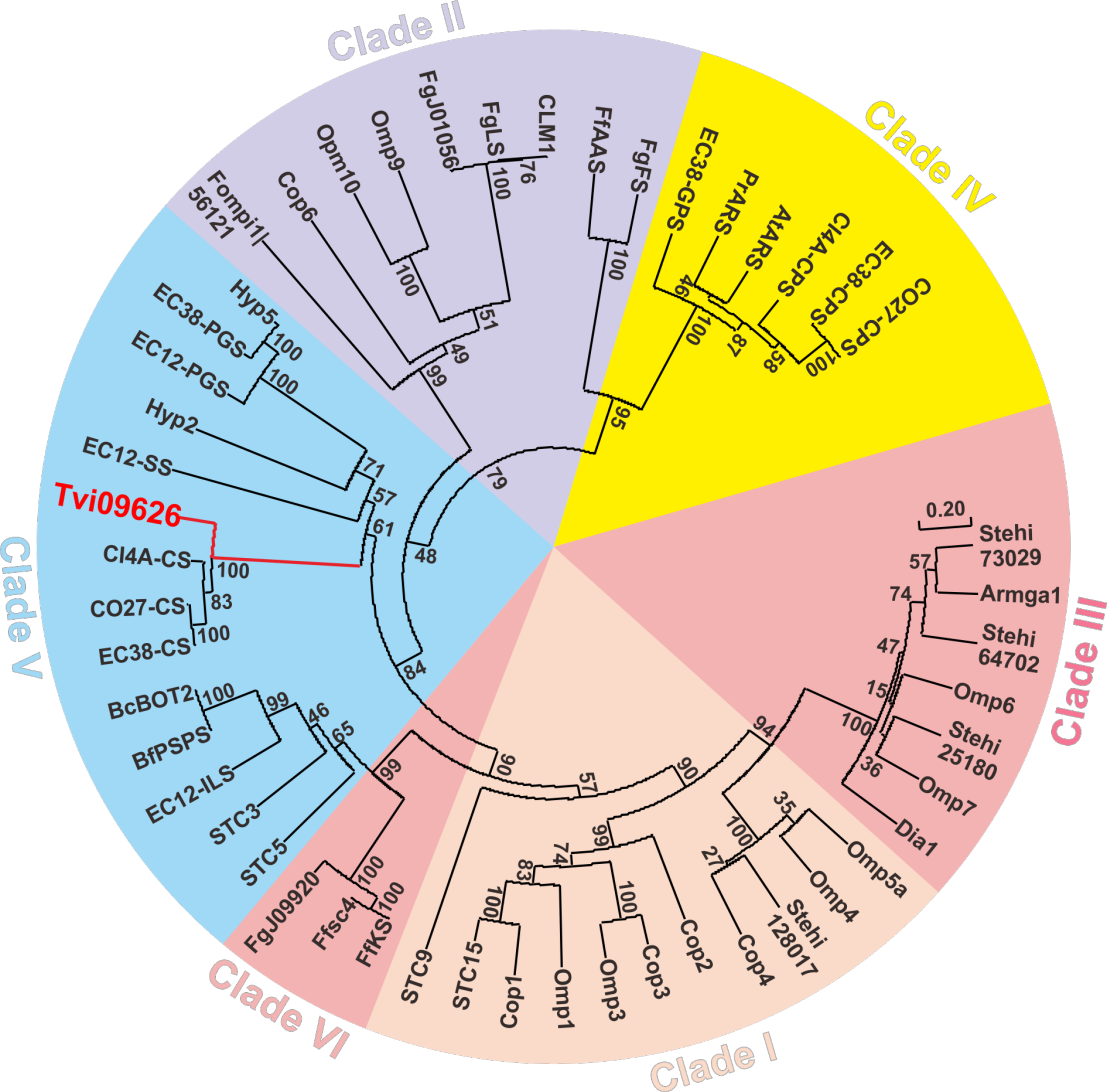
Phylogenetic analysis of Tvi09626 with other characterised terpene synthases. Six clades are marked with different colours and Tvi09626 is labelled in red in Clade V. Percentages indicate branch support based on 1,000 bootstrap replicates.

Through genome sequencing of *T. viride* J1-030 and prediction of the potential terpene synthases in J1-030 genome, gene *Tvi09626* was selected and the following bioinformatics analysis of the function of this unidentified terpene synthase was performed. A protein blast search against the NCBI database was performed with Tvi09626, revealing sequence identities of 89.66% and 85.23% with the enzymes from the strain *T. virens* Gv29-8 [27] and *T. reesei* QM6a [28], respectively, with only predicted functions. Thereafter, an amino acid sequence alignment with several known terpene synthases showed that Tvi09626 had the typical highly conserved ^128^**DDxxD/E** aspartate-rich motif, ^276^**NSE/DTE** triad, ^366^**RY** dimer and ^230^**R** monomer (Figure S1, Supporting Information File 1) [29–31]. Furthermore, in a phylogenetic analysis (Figure 2), Tvi09626 belonged to Clade V of Class I terpene synthases. In previous studies, terpene synthases have been studied in the genus *Trichoderma*, such as trichodiene synthase homologous gene isolation and characterisation in *T. harzianum* [32] and functional identification of terpene synthase vir4 in *T. virens* [33]. However, owing to the general lack of previous studies of terpene synthases in *T. viride*, Tvi09626 is the first terpene synthase well-identified with products characterised in *T. viride*.

### In vitro analysis of Tvi09626 function

**Figure 3:**
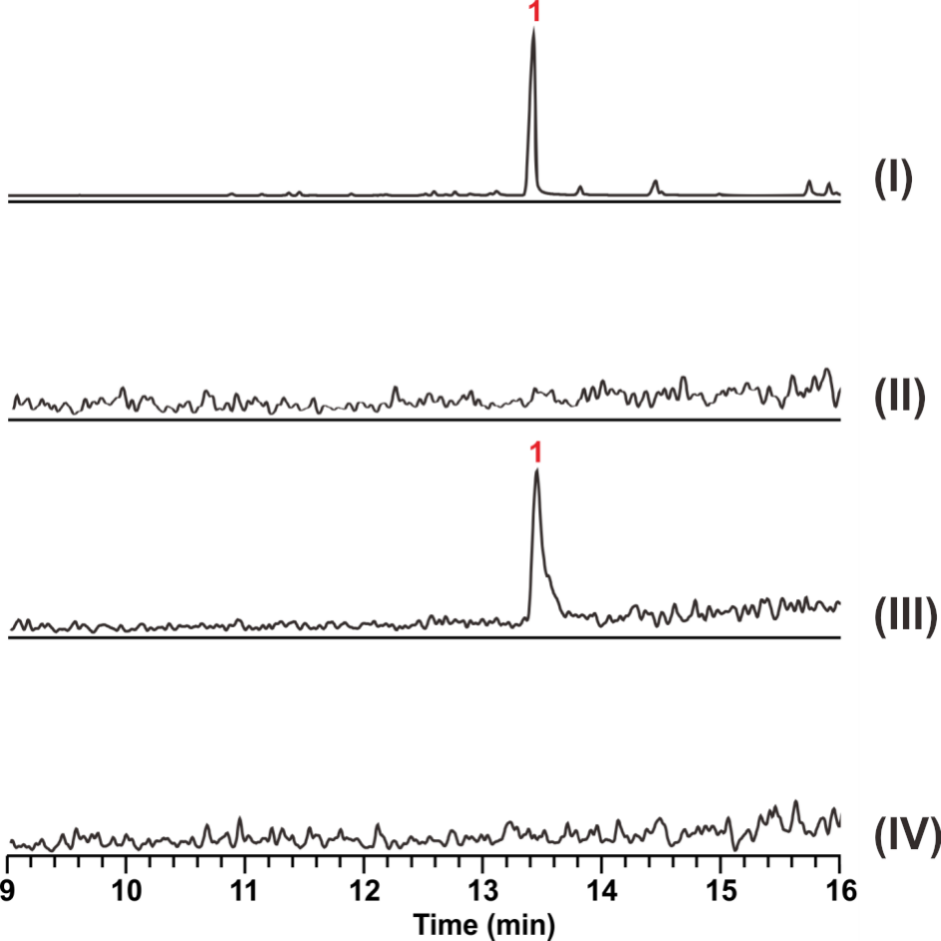
GC–MS chromatogram of products in vivo (I), in yeast YZL141 (II), in vitro Tvi09626 with FPP (III), and boiled Tvi09626 with FPP (IV).

To confirm the function of the candidate enzyme, the DNA sequence of *Tvi09626* was amplified by touchdown PCR from the *T. viride* genome. The gene fragment was cloned into a pET28a (+) vector to construct the plasmid pXS222. Next, pXS222 was transformed into BL21 to overexpress and purify Tvi09626 (Figure S2, Supporting Information File 1). The substrates GPP, FPP and GGPP were incubated with the protein individually and the products were detected and analysed by gas chromatography/mass spectrometry (GC–MS) [30,34]. In vitro assays clearly showed that Tvi09626 could use FPP as its only substrate to produce compound **1** (Figure 3 and Figure S3, Supporting Information File 1).

### Heterologous expression of Tvi09626 in *S. cerevisiae*

**Figure 4:**
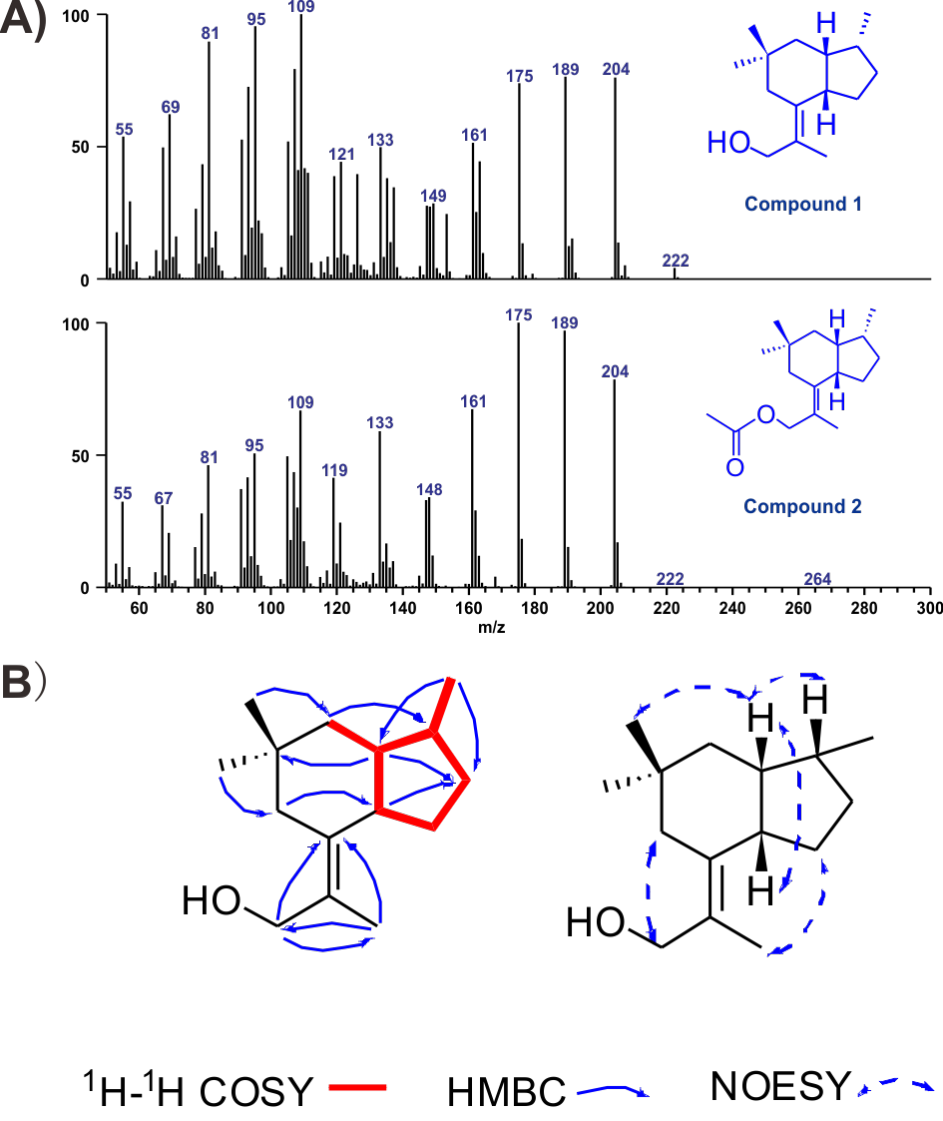
Characterisation of Tvi09626 products. (A) Mass spectra of compound **1** at *t*_R_ = 13.46 min with *m*/*z* 222 and compound **2** at *t*_R_ = 14.53 min with *m*/*z* 264. (B) COSY, HMBC and NOESY correlations for compound **1**.

To further verify the function of the putative terpene synthase, a metabolic engineering strategy was used to reconstruct an FPP overproduction platform in *S. cerevisiae* in order to obtain sufficient quantities of the products of Tvi09626 for chemical structural characterisation. *S. cerevisiae* YZL141, engineered previously [21], was used owing to its ability to provide enough IPP, DMAPP, and FPP for the production of terpenoids (Figure 1). After 72 h of shaken-flask fermentation, the strain was extracted with hexane/ethyl acetate (4:1), pre-separated by silica gel column chromatography, and detected by GC–MS. Similar to the in vitro assay results, compound **1** was the final product (Figure 4). Interestingly, during the extraction process, compound **2** was detected at a retention time of 14.53 min and identified as esterified compound **1** (Figure 4 and Table S4, Supporting Information File 1). Using the metabolic engineering strategy, the product of Tvi09626 was efficiently enriched via an abundant FPP supply.

### Detection and characterisation of Tvi09626 products

By semi-preparative high-performance liquid chromatography (HPLC), we purified compound **1** and compound **2** (23.1 mg and 13.2 mg, respectively). The structures of the two new compounds were characterised by 1D and 2D NMR spectroscopy (Table 1, Table S4, and Figures S4–S15, Supporting Information File 1).

**Table 1:** ^1^H NMR (400 MHz, CDCl_3_) and ^13^C NMR (100 MHz) data for compound **1** in CDCl_3_.

Position	δ_C_	δ_H_

**1**	45.2	1.68 (dd, 8.2, 4.3 Hz, 1H)
**2**	40.6	1.38 (ddd, *J* = 12.8, 3.9, 1.6 Hz, 1H), 1.16 (t, *J* = 12.9 Hz,1H)
**3**	33	—
**4**	43.8	2.35 (dd, *J* = 13.8, 1.4 Hz, 1H), 1.60 (d, *J* = 13.8 Hz, 1H)
**5**	137.4	—
**6**	47.6	1.96 (dd, *J* = 12.3, 6.1 Hz, 1H)
**7**	30.9	2.10 (m, 1H), 1.60 (m, 1H)
**8**	33.3	2.07 (m, 1H), 1.07 (m, 1H)
**9**	31.6	1.99 (m, 1H)
**10**	126.5	—
**11**	65.2	4.18 (d, *J* = 11.6 Hz, 1H),3.97 (d, *J* = 9.8 Hz, 1H)
**12**	17.85	1.87 (t, *J* = 1.2 Hz, 3H)
**13**	32	0.96 (s, 3H)
**14**	26.1	0.83 (s, 3H)
**15**	17.78	0.80 (d, *J* = 7.0 Hz, 3H)
**16**	—	3.47 (s, 1H)

Compound **1** was a new compound with a known skeleton [35], isolated as a white powder. ^1^H and ^13^C NMR data showed four methyl groups at δ_H_ 1.87 (t, *J* = 1.2 Hz, 3H,), δ_H_ 0.96 (s, 3H), δ_H_ 0.83 (s, 3H), and δ_H_ 0.80 (d, *J* = 7.0 Hz, 3H). Five methylenes were detected, including an oxygenated one at δ_H_ 4.18 (d, *J* = 11.6 Hz, 1H), 3.97 (d, *J* = 9.8 Hz, 1H), as well as three methines and three quaternary carbons including a double bond at δ_C_ 137.4 (C-5), 126.5 (C-10). The 2D NMR data indicated that compound **1** is a 5/6 bicyclic sesquiterpene with the molecular formula C_15_H_26_O (Figure 1). Interestingly, compound **1** contained a quaternary carbon with two methyl groups, which is uncommon for the cyclization mechanism of sesquiterpenoids and needs further investigation.

Compound **2** was purified as a white powder. ^1^H and ^13^C NMR data showed chemical shifts of five methyl groups at δ_H_ 1.83 (t, *J* = 1.2 Hz, 3H), δ_H_ 0.96 (s, 3H), δ_H_ 0.82 (s, 3H), δ_H_ 0.80 (d, *J* = 7.0 Hz, 3H), and δ_H_ 2.06 (s, 3H). Five methylenes were identified, including an esterified group at C-11 with a resonance of δ_H_ 4.64 (d, *J* = 11.6 Hz 1H), 4.46 (d, *J* = 11.6 Hz, 1H), three methines, and three quaternary carbons including a double bond at δ_C_ 140.37 (C-5), 122.04 (C-10). Compared with 2D NMR information of compound **1**, compound **2** was a C-11 esterified **1** with the molecular formula C_17_H_28_O_2_ (Table S4, Supporting Information File 1), which may represent the esterification reaction during the extraction process.

The 5/6 bicyclic sesquiterpene identified and characterised in this study was a brasilane-type sesquiterpenoid; this sesquiterpenoid type is typically isolated from cultures of the basidiomycete *Coltricia sideroides* in combination with its two new alkane derivatives colisiderin A and (7*E*,9*E*)-undeca-7,9-diene-2,4,5-triol [35] and from the organic extract of the red alga *Laurencia obtusa* [36]. However, to the best of our knowledge, ours is the first report of these brasilane-type sesquiterpenes obtained via biosynthetic genes.

### Metal ion dependency of Tvi09626 and its kinetics

**Figure 5:**
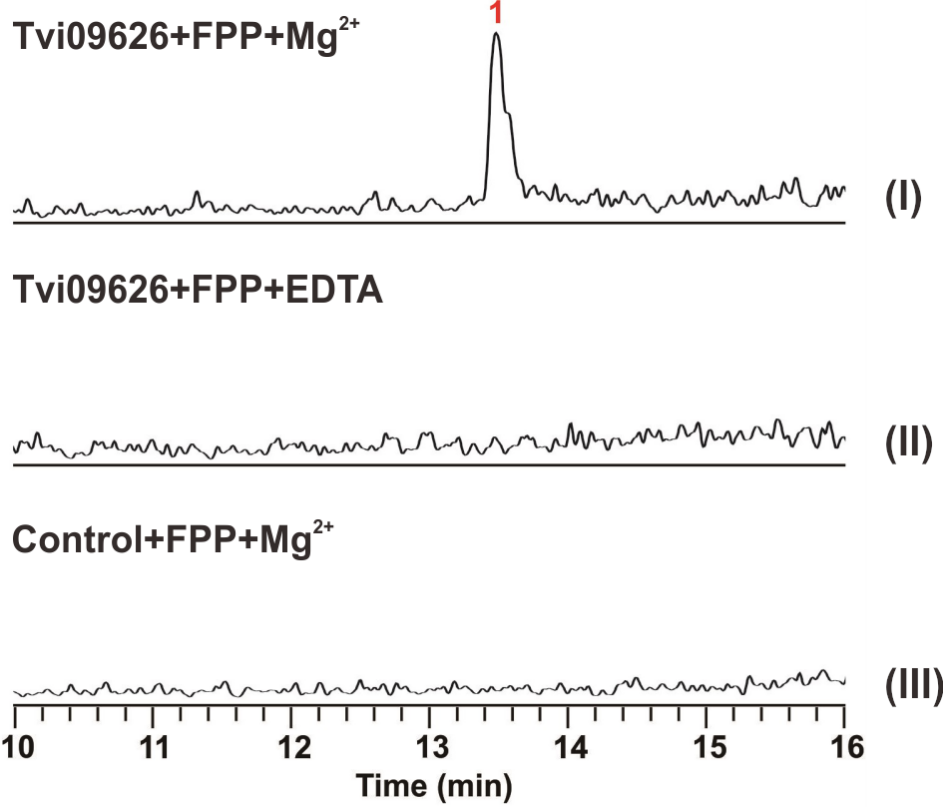
GC–MS chromatogram for the metal ion dependency assay.

As reported previously, most terpene synthases are active in the presence of Mg^2+^ ions [8,37]. To test the Mg^2+^ dependency of Tvi09626, an in vitro assay was performed. The GC–MS analysis showed that in the presence of Mg^2+^, compound **1** can be obtained, whereas without Mg^2+^ or added EDTA (2.5 mM), compound **1** cannot be detected (Figure 5). This assay demonstrated that Tvi09626 was a Mg^2+^-dependent sesquiterpene synthase. In a kinetics analysis, the turnover rate (*k*_cat_) of the enzyme with FPP was (15 ± 0.3) × 10^−2^, which is similar to those of omp6 and omp7. Its substrate affinity (*K*_m_) was (0.44 ± 0.11) × 10^−6^, one-tenth that of omp6 and nearly a quarter that of omp7. The catalytic efficiency (*k*_cat_/*K*_m_) of Tvi09626 was (35.32 ± 0.57) × 10^3^, higher than that of omp6 and lower than that of omp7 [21,38].

## Conclusion

In conclusion, we identified a novel sesquiterpene synthase, Tvi09626, from *T. viride* using a strong sesquiterpene overproduction platform with *S. cerevisiae* YZL141; it is the first biochemically identified and characterised sesquiterpene synthase from this filamentous fungus. In an analysis of its relative structure, the product of this enzyme was characterised as a 5/6 bicyclic sesquiterpene compound **1** oxygenated at C-11. Interestingly, esterified compound **1** was isolated during the product extraction process, suggesting an esterification reaction. It contained a quaternary carbon with two methyl groups, which is uncommon of the cyclization mechanism of sesquiterpenoids and needs to be studied in the future. To the best of our knowledge, this study reports the first use of a biosynthetic gene to obtain a brasilane-type sesquiterpene.

## Supporting Information

File 1 Experimental part and supplementary figures and tables.
