# Supplementary material for: Genome mining in Trichoderma viride J1-030: discovery and identification of novel sesquiterpene synthase and its products
Source: Beilstein J Org Chem. 2019 Aug 28;15:2052–8. doi: 10.3762/bjoc.15.202 (PMC6720227; doi:10.3762/bjoc.15.202)
Supplement: File 1 — Experimental part and supplementary figures and tables. [file Beilstein_J_Org_Chem-15-2052-s001.pdf]

BEILSTEIN JOURNAL OF ORGANIC CHEMISTRY

Supporting Information

for

Genome mining in *Trichoderma viride* J1-030: discovery and identification of novel sesquiterpene synthase and its products

Xiang Sun, You-Sheng Cai, Yujie Yuan, Guangkai Bian, Ziling Ye, Zixin Deng and Tiangang Liu

Beilstein J. Org. Chem. **2019,** *15,* 2052–2058. doi:10.3762/bjoc.15.202

Experimental part and supplementary figures and tables

License and Terms: This is a supporting information file under the terms of the Creative Commons Attribution License (http://creativecommons.org/licenses/by/4.0). Please note that the reuse, redistribution and reproduction in particular requires that the authors and source are credited.
The license is subject to the *Beilstein Journal of Organic Chemistry* terms and conditions: (https://www.beilstein-journals.org/bjoc)

Table of contents

Experimental part of main article **S3–S11**

Supplementary Figures S1–S15 **S12–S25**

Supplementary Tables S1–S4 **S26–S28**

Supplementary references **S29–S30**

Experimental

General procedures

1D and 2D NMR data were recorded using an Agilent (Santa Clara, CA, USA) DD2 (400 MHz or 600 MHz) NMR spectrometer. $CDCl_3$ was used for NMR measurements. Chemical shifts were recorded in ppm downfield from tetramethylsilane and data are noted as follows: chemical shift, multiplicity (s = singlet, d = doublet, t = triplet, q = quartet, m = multiplet, br = broad), coupling constant (Hz), and integration. Column chromatography was performed with an 80–100 mesh silica gel. All salts and chemical reagents were purchased from Shenshi Chemical Reagent Co. Ltd (Wuhan, China).

Strains and media

The details of the strains used in this study are listed in Table S1. *Escherichia coli* strain DH10B was used for cloning and propagating all plasmids. *E. coli* strain BL21 (DE3) was used for heterologous protein expression. *S. cerevisiae*

YZL141 was constructed as a platform for providing the precursors IPP and DMAPP for the heterologous expression of Tvi09626 and over production of its sesquiterpene product. *T. viride* J1-030 was cultivated in PDA medium [1].

Bioinformatic analysis of the predicted terpene synthase Tvi09626

To predict terpene synthases in *T. viride* J1-030, a multiple sequence alignment was obtained using CLUSTAL W 2.0.12 [2-4]. Evolutionary analyses were performed using MEGA7 [5]. A phylogenetic tree was constructed using the maximum likelihood method based on the Jones-Taylor-Thornton (JTT) matrix-based model. Branch separations reproduced in fewer than 50% of bootstrap replicates were collapsed. The percentage of replicate trees in which the associated taxa clustered together in the bootstrap test (1000 replicates) were shown next to branches. For the phylogenetic analysis of terpene synthases in *T. viride* J1-030, 50 amino acid sequences were used. All positions containing gaps and missing data were eliminated. There were 209 positions in the final dataset.

Plasmid and mutant construction

The sequences of primers used in this study are listed in Supplementary Table S2. Strains and plasmids are summarised in Supplementary Tables S1 and S3. To construct the plasmid expressing sufficient levels of the precursors IPP and DMAPP in *S. cerevisiae*, pYZL141 was constructed by previously described methods [6]. To heterologously express *Tvi09626* in *E. coli*, the coding sequence of *Tvi09626* was amplified from the genome of *T. viride* J1-030 using the primer pair P5/P6, and the fragment was cloned into pET28a(+) by Gibson assembly to reconstitute pXS222 [7]. To confirm the function of Tvi09626 in *S. cerevisiae*, pXS217 was constructed according to the following procedure. *Tvi09626* was amplified from pXS222 using the primer pair P1/P2. The backbone of the plasmid pYeast3939 (constructed by Guangkai Bian) was amplified using the primer pair P3/P4. Finally, two fragments were assembled by Gibson assembly to generate pXS217. The plasmid pYZL141 was linearised and inserted into the GAL1710 site of *S. cerevisiae* CENPK2-1D to generate the mutant *S. cerevisiae* YZL141. The plasmid pXS217 was linearised and

inserted into the HIS3 site of *S. cerevisiae* YZL141 to generate *S. cerevisiae* XS217.

Expression and purification of Tvi09626

Plasmid pXS222 was transformed into *E. coli* strain BL21 (DE3) and cultivated in 2 L flasks including 1 L of LB medium at 37 °C supplemented with 50 mg/L kanamycin (KAN). Thereafter, 0.1 mM isopropyl β-D-1-thiogalactopyranoside (IPTG) was added to cultures when the OD_{600} reached 0.7–0.8, followed by cultivation for another 18 h at 16 °C. The cells were harvested and resuspended in 25 mL of buffer A (50 mM Tris-Cl, 300 mM NaCl, 4 mM β-mercaptoethanol, pH 7.6). Cell lysis was performed using a high-pressure homogeniser at 10500–12000 psi, followed by centrifugation at 35,000*g* for 30 min. The supernatant was collected and filtered with a 0.45 μm filter membrane, buffer B was added, and the supernatant was adjusted with 30 mM imidazole to reduce miscellaneous nonspecific protein binding. Ni-NTA affinity chromatography column (GE Healthcare, Little Chalfont, UK) was applied to purify the his-tagged protein. The heterologously expressed protein was desalinised,

concentrated, and stored in Tris buffer (100 mM Tris base, 12% glycerol, pH 7.6) for subsequent in vitro assays.

The protein concentration was detected using a BCA Protein Quantification Kit (Thermo Fisher Scientific, Waltham, MA, USA) and recorded using a Thermo Multimode Plate Reader (Thermo Fisher Scientific).

In vitro enzyme assays and kinetic measurements

To test the terpene synthase activity of Tvi09626, an in vitro assay was performed as described in previous studies [3,8]. To confirm the substrate selectivity of Tvi09626, GPP, FPP, and GGPP were selected. The in vitro reactions were performed in a 200 μL system and executed with 10 μM Tvi09626, 2 mM Mg^{2+}, 100 μM substrates (GPP, FPP, or GGPP), 10% glycerol and ddH_2O at 30 ℃ overnight. The reaction products were extracted with 250 μL of hexane and detected by GC–MS (Figure S3).

Enzyme reactions were performed in a 100 μL system with 1 mg/mL Tvi09626, 50 μL of pyrophosphate reagent, 2 mM Mg^{2+}, 10% glycerol, and 1–100 mM FPP. The reaction was recorded based on the release of pyrophosphate (PPi) using

a Thermo Multimode Plate Reader, as described previously [6,9].

Functional characterisation of Tvi09626 in *S. cerevisiae*

S. cerevisiae XS217 was activated on a YPD ager plate and inoculated in 5 mL of YPD (2% glucose) medium at 30 ℃ overnight. Thereafter, 1% of the culture was transformed into a 250 mL shaken-flask with 50 mL of YPD (2% glucose) at 30 ℃ overnight. Finally, 1% of the culture was incubated into a 2 L shake flask with 1 L of YPD (2% glucose and 1% D-(+)-galactose) at 30 ℃ for 3 days of fermentation. The strains were collected and the products were extracted with hexane/ethyl acetate (4:1) four times. The organic layer was combined, concentrated using a rotary evaporator, and re-dissolved with hexane for GC/MS and follow-up experiments.

Isolation and structural elucidation of compounds

The re-dissolved extracts were pre-separated by silica gel (80–100 mesh, 20 g) column chromatography with petroleum ether (PE)/ethyl acetate (EAc) (100:1 to 1:1). Fractions were collected and analysed by GC–MS for identification. Final purification was performed by preparative HPLC using an

Ultimate 3000 HPLC equipped with a Waters Xterra RP C18 column (3.9 × 150 mm, 5 μm). The elution gradient was 0–20 min: 90–100% methanol, 20–40 min: 100% methanol, 40–40.05 min: 100–90% methanol, 40.05–42 min: 90% methanol, ultraviolet (UV) light at λ = 210 nm, yielding compounds **1** and **2** with different fragments. Product structures were detected by GC–MS and 1D and 2D NMR.

Reagents and chemicals

All commercial reagents were obtained as follows.

PrimeSTAR GXL DNA Polymerase was purchased from Takara Bio (TAKARA, Dalian, China). The Plasmid Mini Kit, Polymerase Chain Reaction (PCR) Purification Kit, and Gel Extraction Kit were purchased from Axygen (Hangzhou, China). Tryptone and yeast extracts for yeast fermentation were purchased from Angel Yeast (Wuhan, China).

The cDNA sequence of *Tvi09626*

ATGGATCAACTCAGGAGTCTCGTCTTTTTTTTACGTGATGTTGCTTTGAAG

CACACACTTGGCACTAGCCATACCAAGAATGATACCACCGAGATATTTGAT

GAACGTAAAGAAGTCATAAAGAGAGTCTTGAATAAGAAAGTTCTCGTACCA

GATATTCTTGCTCTCATGCCTGCCTGGCTTAGCGAATTCCAACCAGATATT

GATGAAATCAACTTGGAAATTGACGAATGGCTGAAAACCGTCAATGTCGC

AGAGGAGAAGAAAGCTAAGCATCGAGCCCGCGGCAACTATACCCTTCTAA

CGGGCATTTACTATCCTCACTGCAAGAAGGACAAGATGCTGGTGCTTTCA

CAGTTTCTTTACTGGATATTCTTTTGGGACGATGAAATTGACACTGGGGGT

GAGCTCACCCACGACAGAGAAGGCACACTACAATGTTGCGCTGAAACTC

ACAAGTGTATCGATGACTGTCTCGGCCCCAATCCCAATTACACACCACCA

CCAGGCTCTCGAGGTACTGTGGAAATGTTCTATCCCATTCTCCGAGATCT

TCGAAAGGGTCTTGGACCTGTTTCAACTGAACGTCTCAGGAAGGAGCTG

CATGATTACGTGTCCGGTGCATCTACGCAGCAGGCTGTGAGAGAGGCAG

ATCATCTCCCAGATCCTTGGGATCACTTCCAGATGCGAGCGGACGACGTC

GGTGTTATCCCTTCTATCACGCAAAACGAATTTGCAATGGAGTTTGAGCTC

CCGGAGTGGGTGCGCAGGCACGAAGCTATGGAGGAGATTGTCTTGGAG

TGTACCAAGTTGACGATTCTTGTCAACGAGGTCCAAAGCTCCCAGAAAGA

ATTTCGCGTTTCACAGCTCGAGAACCTTTGCTTCCTCTTCATGAACACAAA

CAACCTATCGATTGAAGAGGCCATAGACAAGGTTCTTGGTATCTTAAAAGA

GCATTATGAGATTTGTGTGGCTGCGGAGGCCAGGCTTCCTTGGAGCAAA

ACTGACGAGAAATTTAACGAGGATCTCCGCGAGTATGTACGCGGATGTCA

AAGACTAGCGACGGGCACCGCATGCTGGAGCTACAACTGTGAGAGATAT

TTTAAGCTGAGCCAGGTGAATGATAAGCGAGAATTATTGTTGGATTACTCA

TACCAAAAATAG

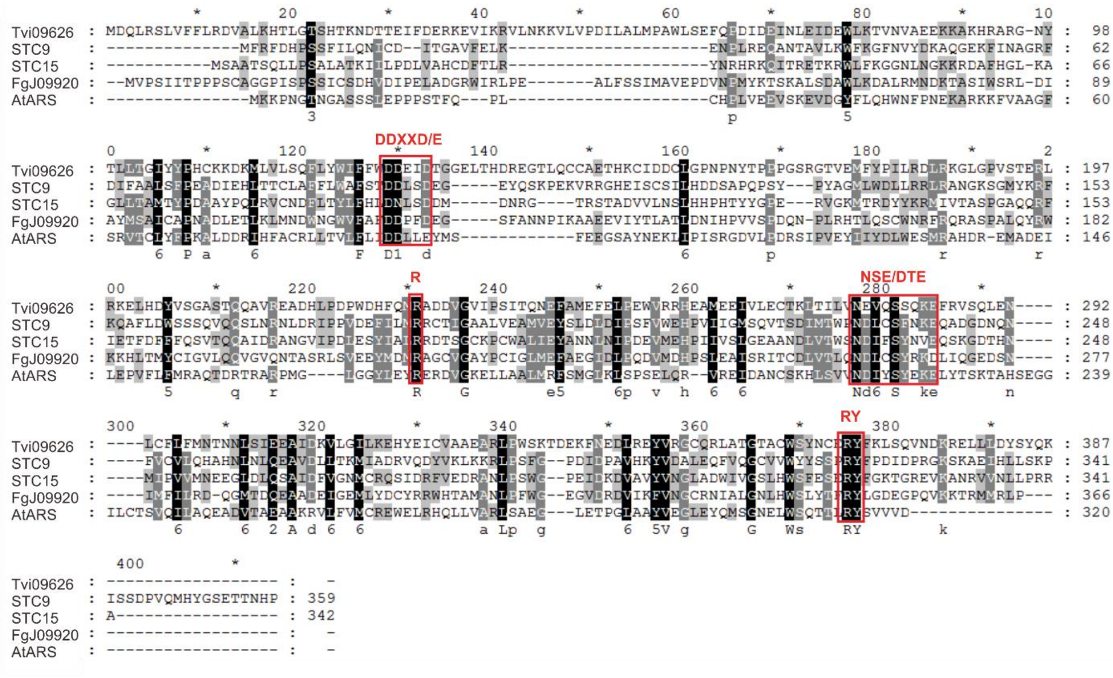

Figure S1: Amino acid sequence alignments of Tvi09626 and previously characterised sesquiterpene synthases. Red boxes indicate the conserved **DDXXD/E**, **NSE/DTE**, **R,** and **RY** motifs.

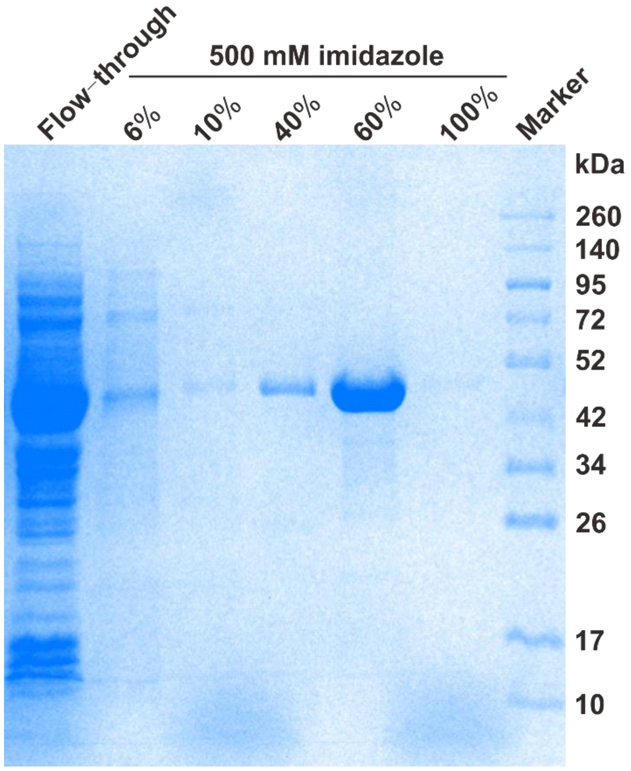

Figure S2: SDS-PAGE gel of protein Tvi09626. Target purified protein showed a single band with a molecular weight of 47.3 kDa.

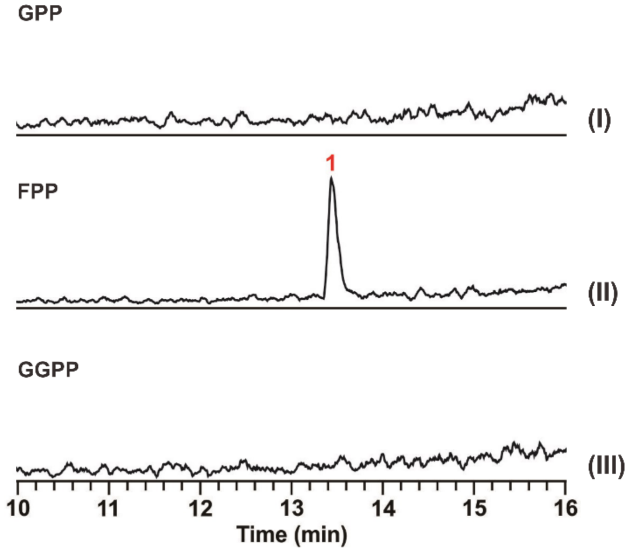

Figure S3: Incubation of Tvi09626 with GPP, FPP and GGPP.

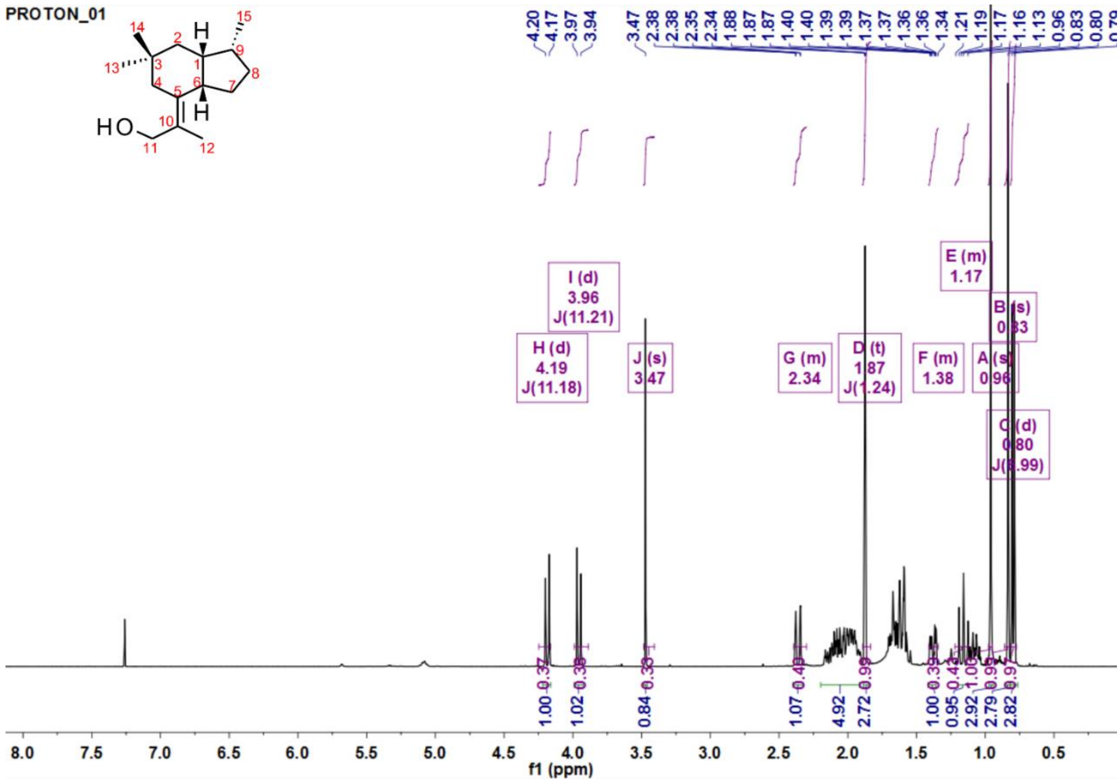

Figure S4: ^{1}H NMR spectrum of compound **1** ($CDCl_3$, 400 MHz).

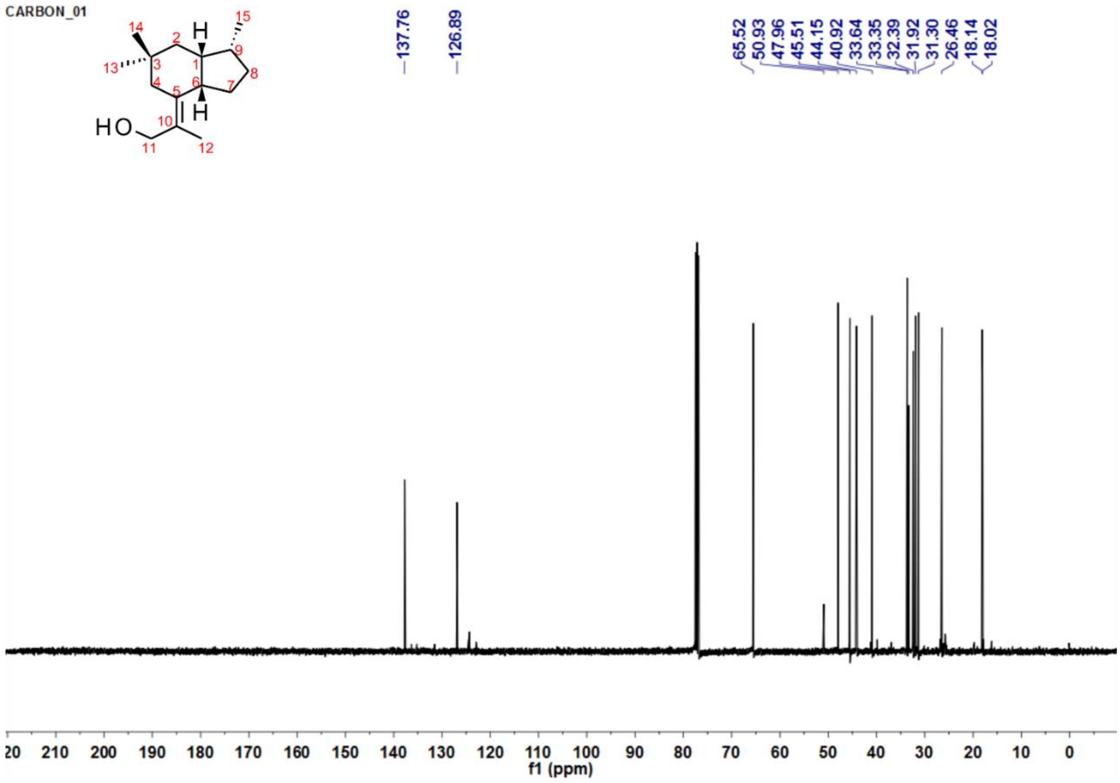

Figure S5: ^{13}C NMR spectrum of compound **1** ($CDCl_3$, 100 MHz).

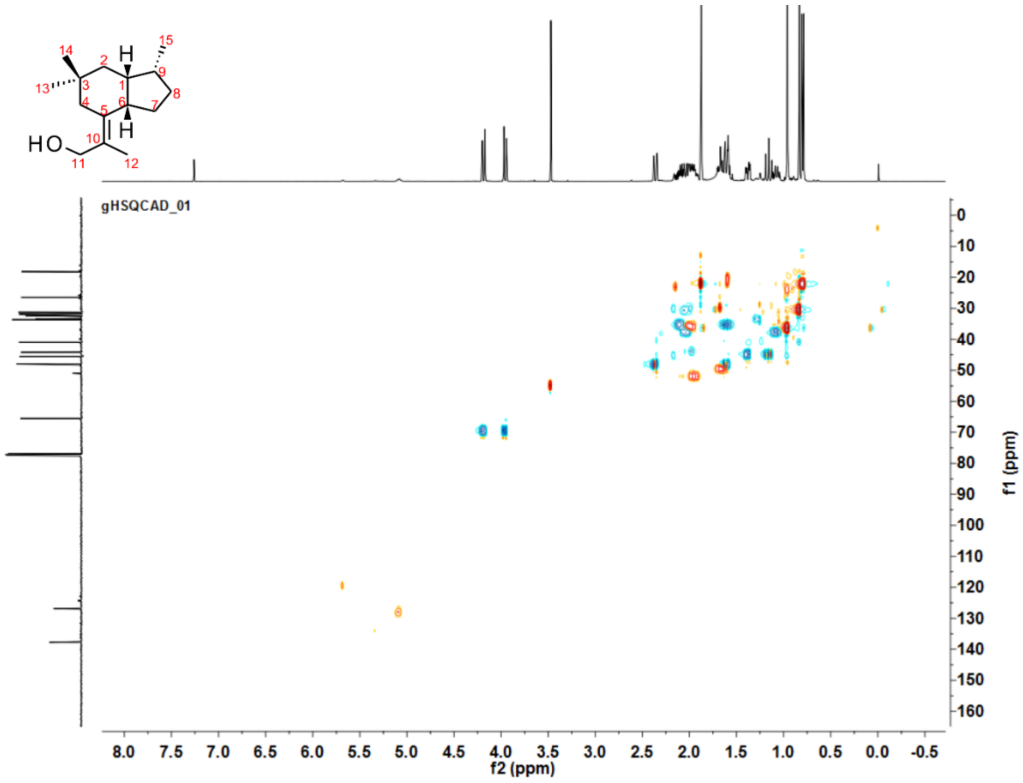

Figure S6: HSQC spectrum of compound **1** in $CDCl_3$.

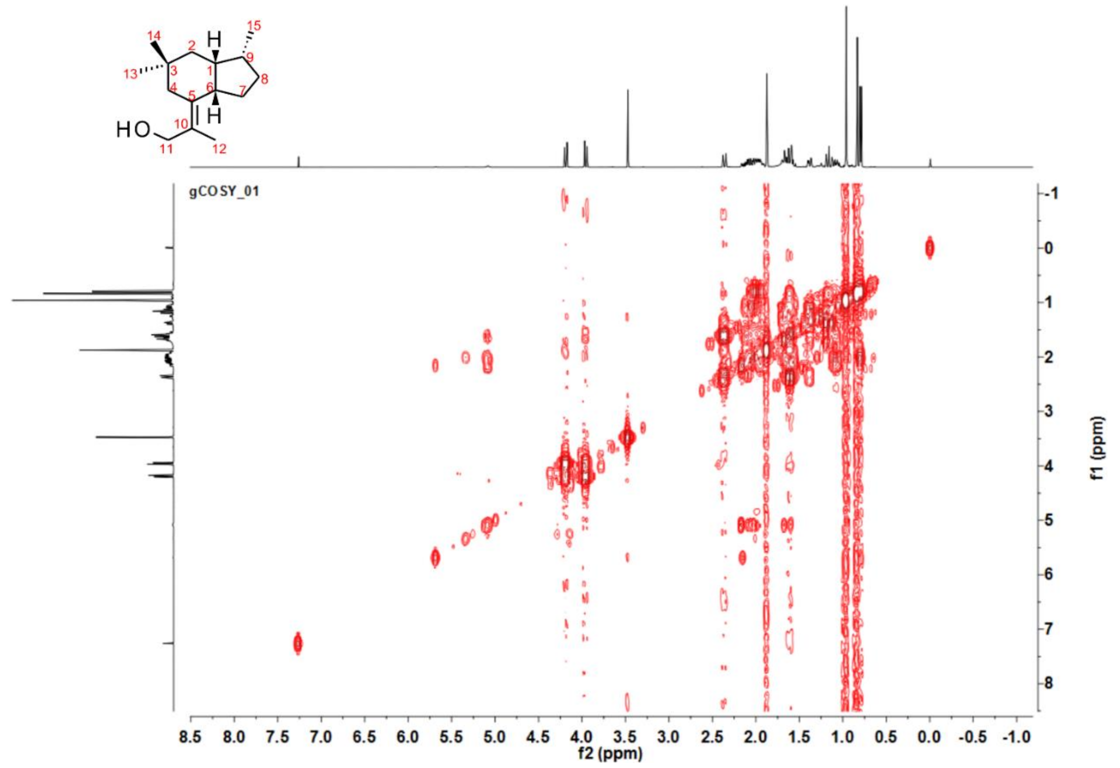

Figure S7: ^1H-^1H COSY spectrum of compound **1** in $CDCl_3$.

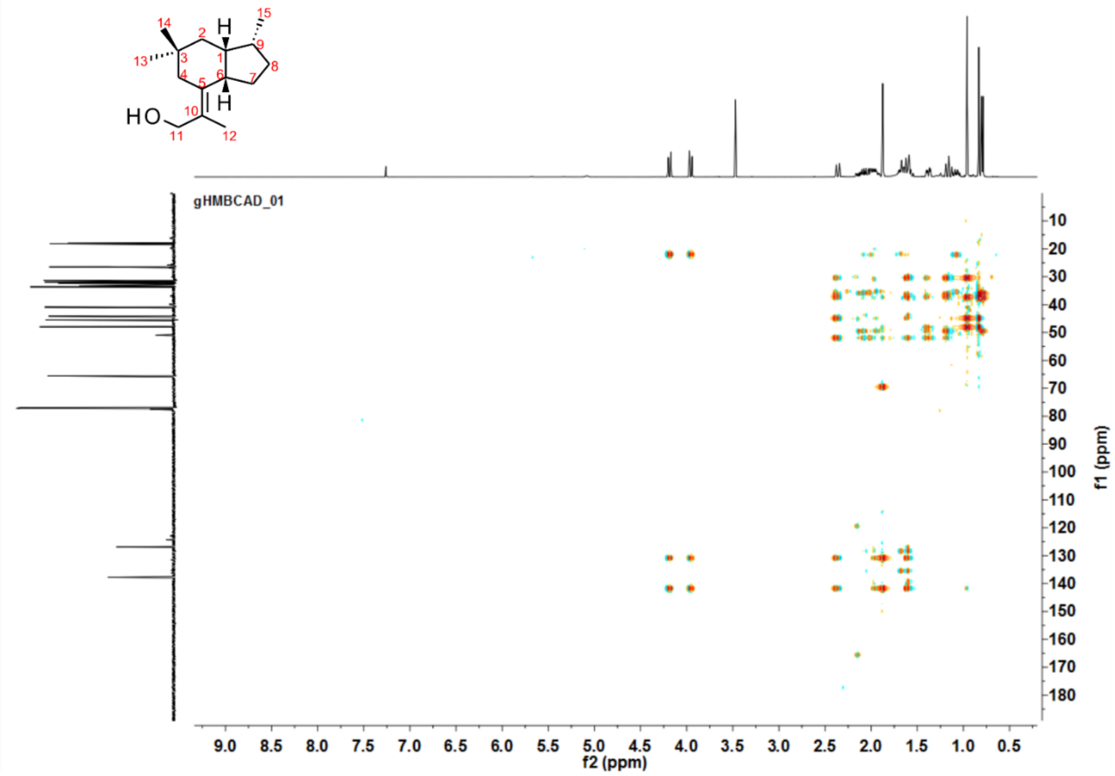

Figure S8: HMBC spectrum of compound **1** in $CDCl_3$.

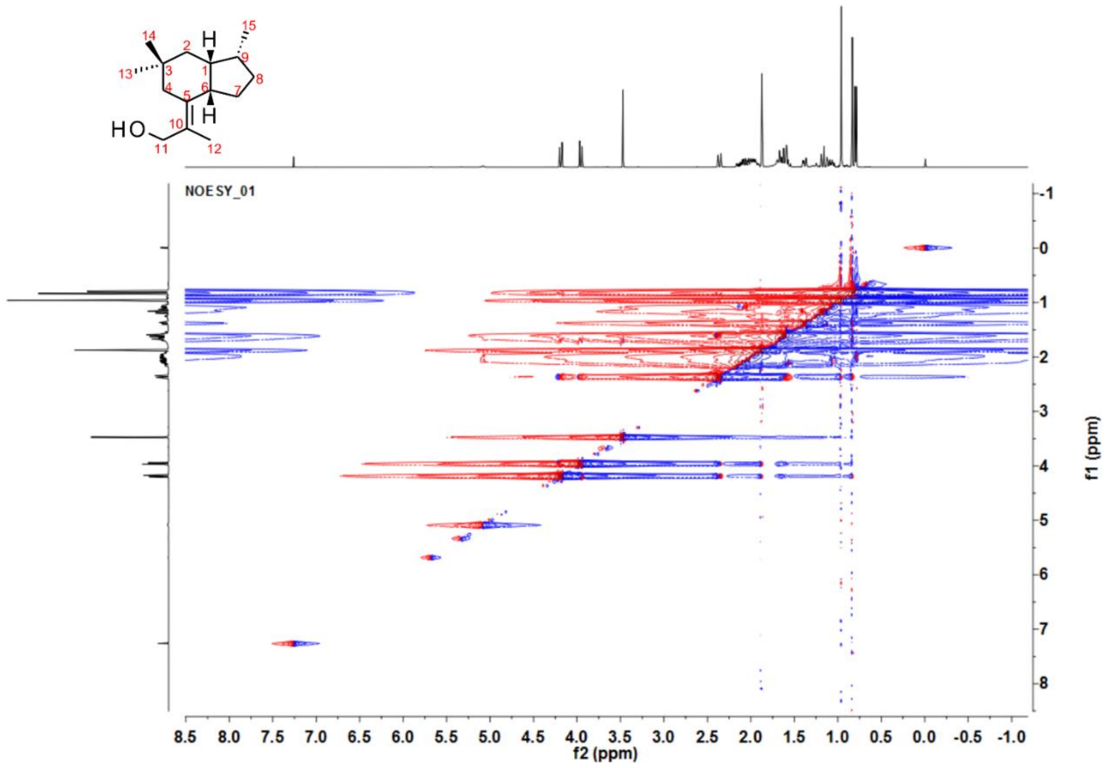

Figure S9: NOESY spectrum of compound **1** in $CDCl_3$.

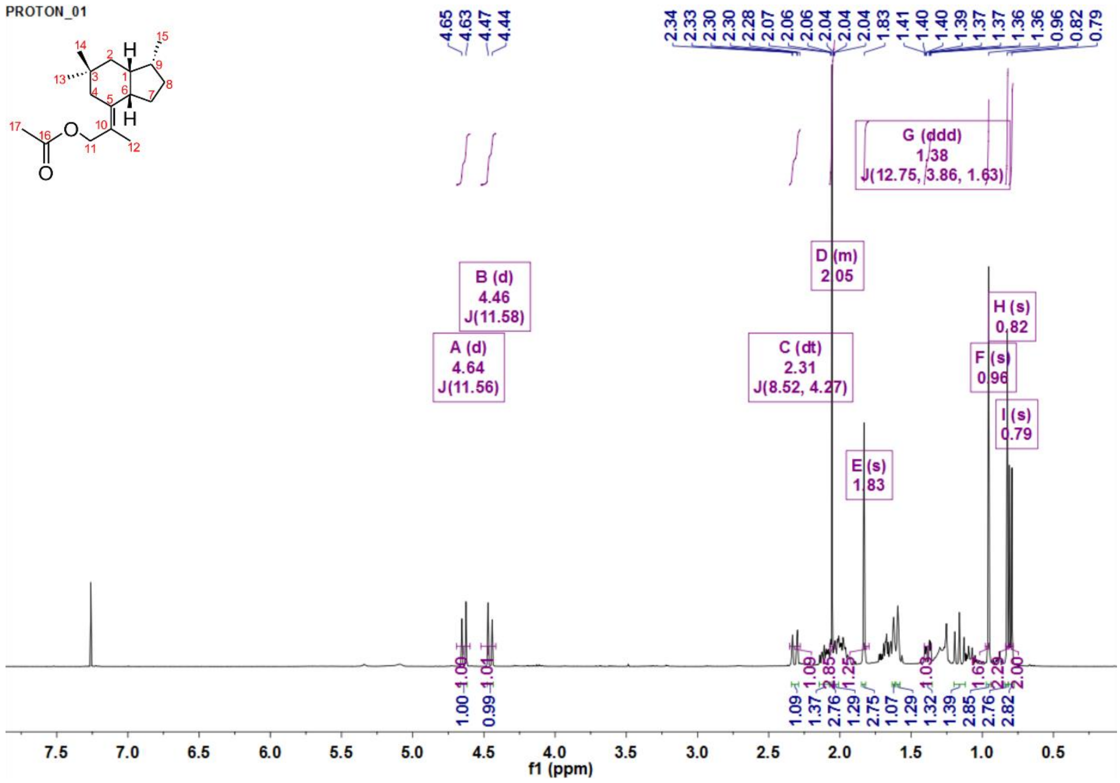

Figure S10: ^{1}H NMR spectrum of compound **2** ($CDCl_3$, 400 MHz).

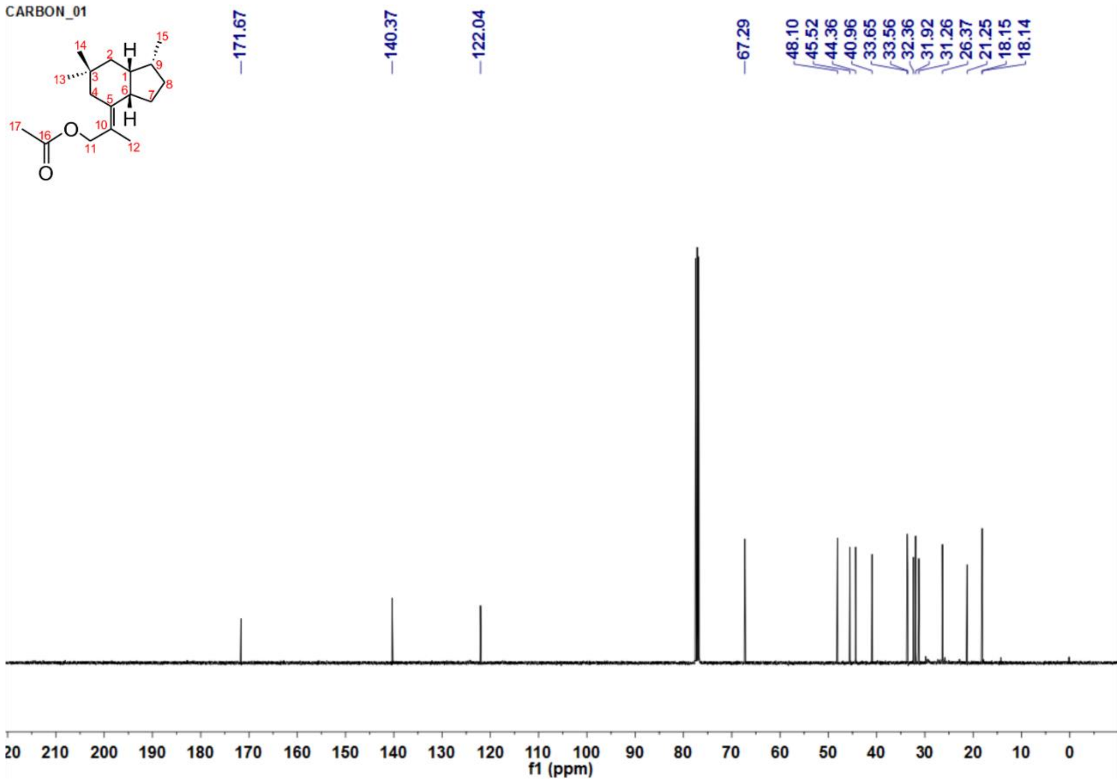

Figure S11: ^{13}C NMR spectrum of compound **2** ($CDCl_3$, 100 MHz).

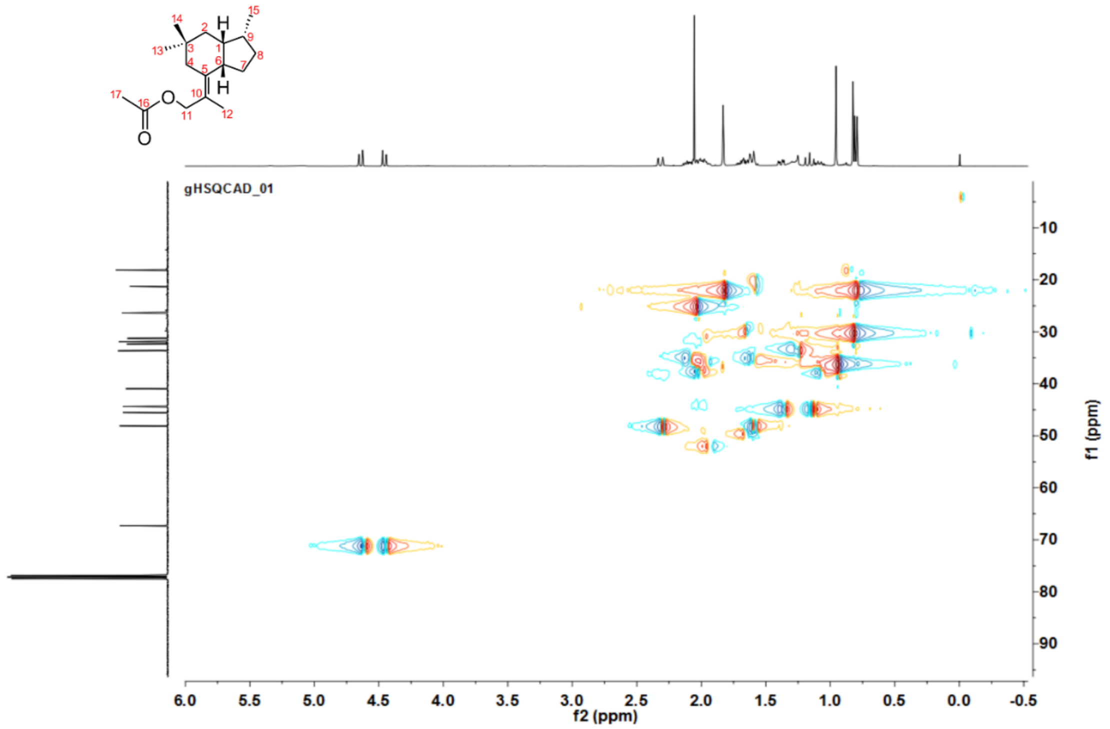

Figure S12: HSQC spectrum of compound **2** in $CDCl_3$.

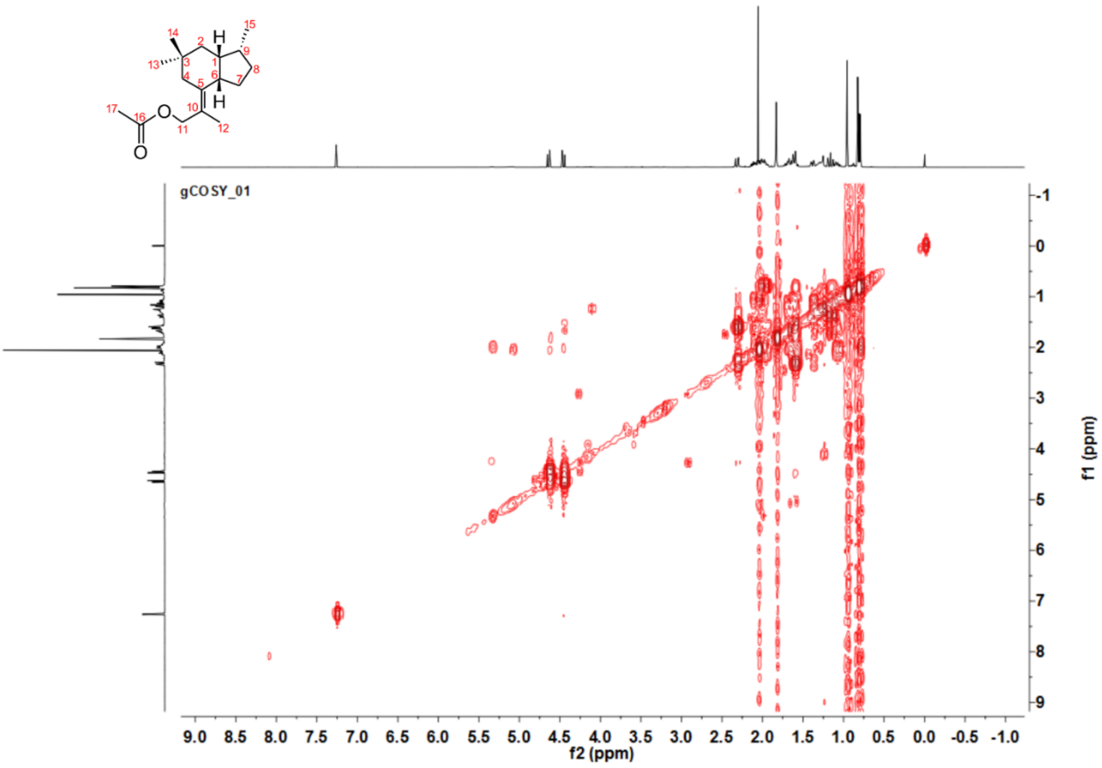

Figure S13: ^{1}H-^{1}H COSY spectrum of compound **2** in $CDCl_3$.

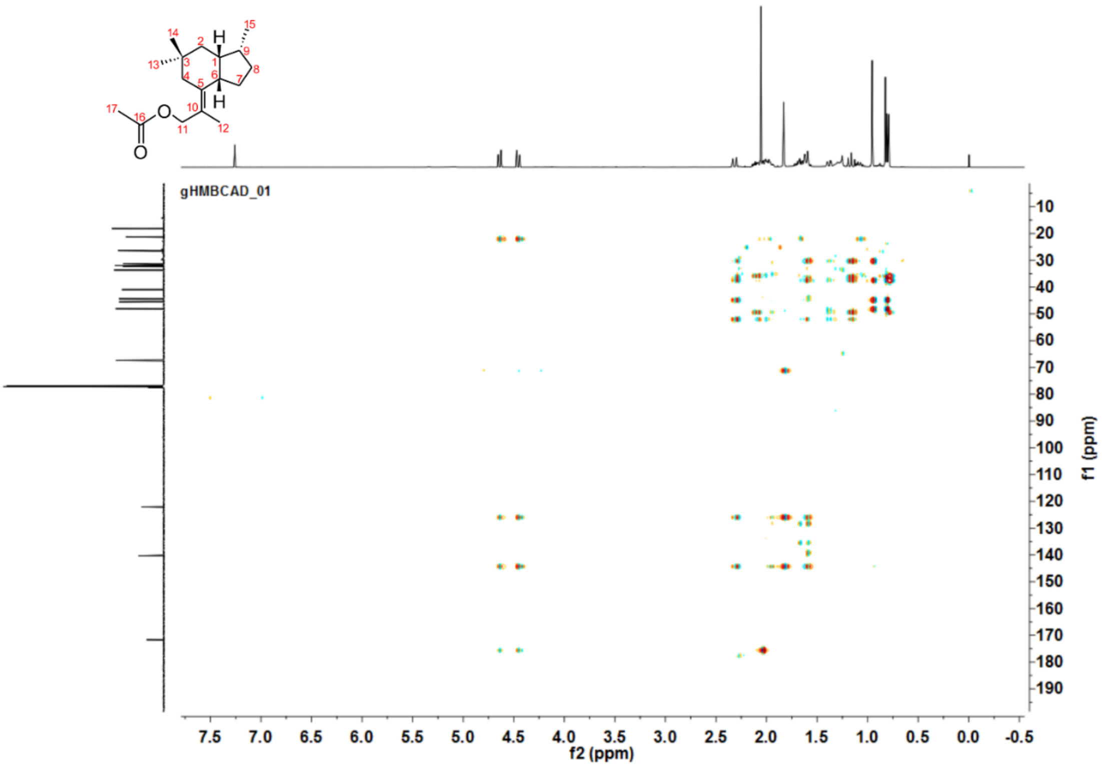

Figure S14: HMBC spectrum of compound **2** in $CDCl_3$.

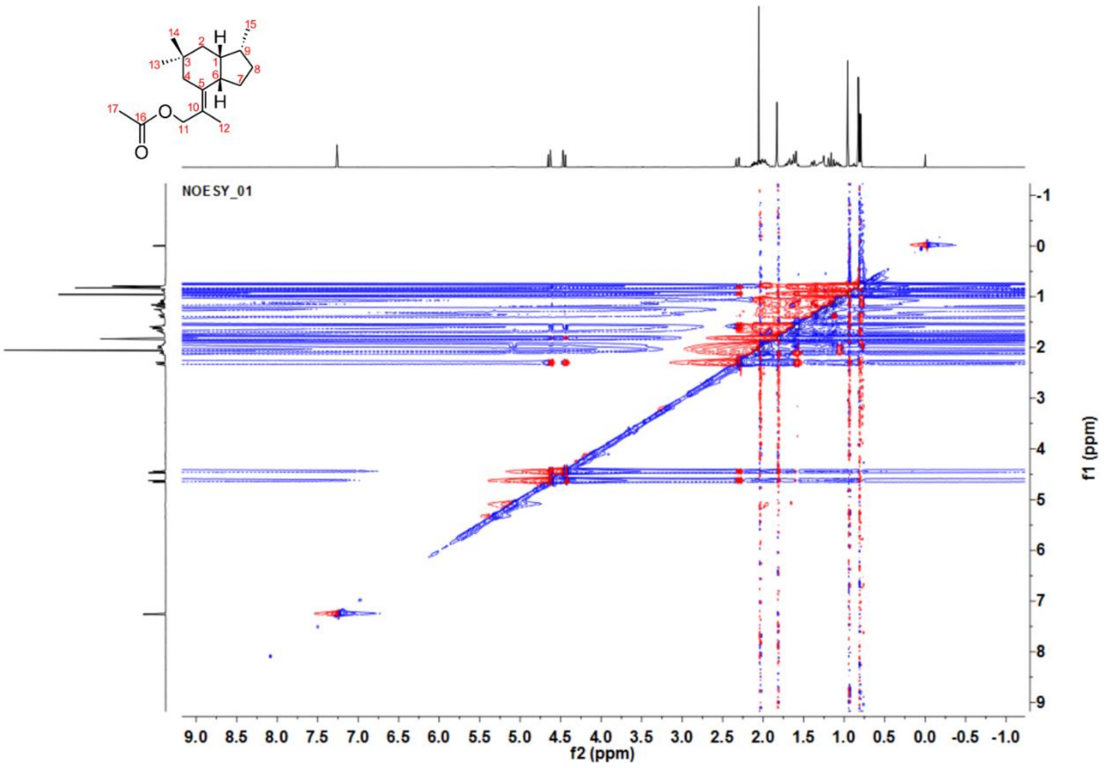

Figure S15: NOESY spectrum of compound **2** in $CDCl_3$.

Table S1: Details of strains used in this study.

Strains	Relevant genotype	Reference
BL21 (DE3)	*E. coli B F⁻dcm ompT hsdSB(rB⁻ mB⁻)gal*	Invitrogen
DH10B	*E. coli D F⁻mcrA Δ(mrr-hsdRMS-mcrBC) Φ80dlacZ ΔM15 ΔlacX74 endA1 recA1 deoR Δ(ara,leu)7697 araD139 galU galK nupG rpsL λ⁻*	Invitrogen
CEN.PK2-1D	*Saccharomyces cerevisiae* MATalpha; his3D1; leu2-3_112; ura3-52; trp1-289; MAL2-8c; SUC2	EUROSCARF
YZL141	*S. cerevisiae*:: pGAL10-tHMG1	6
XS217	*S. cerevisiae*:: P_{GAL10}-*tHMG1*; P_{GAL10}-*Tvi09626*; P_{GAL1}-*ERG20*	This work
XS222	*E. coli* BL21:: pXS222	This work

Table S2: Details of primers used in this study.

No.	Primers	Sequence 5'-3'
P1	pXS221-Gene-F	GAGCCGCGCGGCAGCCATATGGATCAACTCAGGAGTCTCG
P2	pXS221-Gene-R-re	CTCGAGTGCGGCCGCAAGCTTCTATTTTTGGTATGAGTAATCCAAC
P3	pXS221-B-F-re	GGATTACTCATACCAAAAATAGAAGCTTGCGGCCGCACTCGAGCAC
P4	pXS221-B-R	CGAGACTCCTGAGTTGATCCATATGGCTGCCGCGCGGCACCAGG
P5	pXS217-Gene-F	ATAAATCATAAGAAATTCGCCTATTTTTGGTATGAGTAATCCAAC
P6	pXS217-Gene-R	AATTTTTGAAAATTCAATATAAGCCACCATGGATCAACTCAGGAGTCTC
P7	pXS217-B-F	GAGTTGATCCATGGTGGCTTATATTGAATTTTCAAAAATTC
P8	pXS217-B-R	GTTGGATTACTCATACCAAAAATAGGCGAATTTCTTATGATTTATG

Table S3: Details of plasmid used in this study.

Plasmids	Description	Reference
pXS217	p426gal derived, *URA*, T_{CYC1}-ERG20-P_{GAL1}-P_{GAL10}-*Tvi09626*-T_{ADH1}	This work
pXS222	pET28a derived, P_{T7}: N-terminal his_6-tag *Tvi09626*	This work
pYeast3939	p426gal derived, *URA*, T_{CYC1}-ERG20-P_{GAL1}-P_{GAL10}-FgJ03939-T_{ADH1}	6

Table S4: ^{1}H NMR (400 MHz, $CDCl_3$) and ^{13}C NMR (100 MHz) Data of compound **2** in $CDCl_3$.

Position	δ_C	δ_H
1	45.52	1.68 (dd, 8.2, 4.3 Hz, 1H)
2	40.96	1.38 (ddd, 12.8, 3.9, 1.6 Hz, 1H), 1.16 (t, 12.9 Hz, 1H)
3	33.56	—
4	44.36	2.32 (dd, 13.8, 1.4 Hz, 1H), 1.59 (m, 1H)
5	140.37	—
6	48.1	1.95 (dd, 12.3, 6.1 Hz, 1H)
7	31.26	2.13 – 2.07 (m, 1H), 1.63 (dd, 9.2, 2.1 Hz, 1H)
8	33.65	2.03 (m, 1H), 1.08 (ddd, 9.6, 7.1, 5.2 Hz, 1H)
9	31.92	1.95 (m, 1H)
10	122.04	—
11	67.29	4.64 (d, 11.6 Hz, 1H), 4.46 (d, 11.6 Hz, 1H)
12	18.15	1.83 (t, 1.2 Hz, 3H)
13	32.36	0.96 (s, 3H)
14	26.37	0.82 (s, 3H)
15	18.14	0.80 (d, 7.0 Hz, 3H)
16	171.67	—
17	21.25	2.06 (s, 3H)

References

1. Bian, G. K.; Rinkel, J.; Wang, Z. Q.; Lauterbach, L.; Hou, A. W.; Yuan, Y. J.; Deng, Z. X.; Liu, T. G.; Dickschat, J. S. *Angew Chem Int Ed Engl*. **2018,** *57*, 15887-15890. doi: 10.1002/anie.201809954.

2. Yuan, Y. J.; Martin L.; Shu, C.; Bian, G. K.; Hu, B.; Yan, P.; Cai, Y. S.; Deng, Z. X.; Rita, B.; Liu, T. G. *ChemBioChem*. **2018,** *20*, 677-682. doi: 10.1002/cbic.201800670.

3. Burkhardt, I.; Siemon, T.; Henrot, M.; Studt, L.; Rösler, S.; Tudzynski, B.; Christmann, M.; Dickschat, J. S. *Angew Chem Int Ed Engl*. **2016,** *55*, 8748-51. doi: 10.1002/anie.201603782.

4. Burkhardt, I.; Kreuzenbeck, N. B.; Beemelmanns, C.; Dickschat, J. S. *Org Biomol Chem*. **2019,** 17, 3348-3355. doi: 10.1039/c8ob02744g.

5. Kumar, S.; Stecher, G.; Tamura, K. *Mol Biol Evol*. **2016,** *33*, 1870-4. doi: 10.1093/molbev/msw054.

6. Bian, G. K.; Hou, A. W.; Yuan, Y. J.; Hu, B.; Cheng, S.; Ye, Z. L.; Di, Y. T.; Deng, Z. X.; Liu, T. G. *Org Lett*. **2018,** *20*, 1626-1629. doi:

10.1021/acs.orglett.8b00366.

7. Gibson, D. G.; Young, L.; Chuang, R. Y.; Venter, J. C.; Hutchison, C. A. 3rd.; Smith, H. O. *Nat Methods*. **2009,** *6*, 343-5. doi: 10.1038/nmeth.1318.

8. Zhu, F. Y.; Zhong, X. F.; Hu, M. Z.; Lu, L.; Deng, Z. X.; Liu, T. G. *Biotechnol Bioeng*. **2014,** *111*, 1396-405. doi: 10.1002/bit.25198.

9. Wawrzyn, G. T.; Quin, M. B.; Choudhary, S.; López-Gallego, F.; Schmidt-Dannert, C. *Chem Biol*. **2012,** *19*, 772-83. doi: 10.1016/j.chembiol.2012.05.012.
